# Inhibition of AEBP1 predisposes cisplatin-resistant oral cancer cells to ferroptosis

**DOI:** 10.1186/s12903-022-02503-9

**Published:** 2022-11-09

**Authors:** Qianwen Zhou, Xiaoqi Wang, Yingxue Zhang, Lie Wang, Zhijun Chen

**Affiliations:** 1 Department of Stomatology, PuRen Hospital, Wuhan University of Science and Technology, No.1, Benxi Street, Hongwei Road, Qingshan District, Wuhan, 430081 Hubei China; 2 Department of Stomatology, The First Affiliated Hospital of Soochow University, No.188, Shizi Street, Gusu District, Suzhou, 215000 Jiangsu China; 3 Department of Stomatology, Maternal and Child Health Hospital of Hubei Province, No.745 Wuluo Road, Hongshan District, Wuhan, 430070 Hubei China

**Keywords:** Ferroptosis, AEBP1, Cisplatin-resistant, Oral cancer

## Abstract

**Background:**

Studies have shown that excessive iron can lead to an increased incidence of cancer. The role of adipocyte enhancer-binding protein 1 (AEBP1) on ferroptosis is unknown. Thus, we explored the effect of AEBP1 silencing in regulation of ferroptosis in cisplatin-resistant oral cancer cells.

**Methods:**

The functions of AEBP1 silencing and sulfasalazine (SSZ) treatment were determined on oral cancer cell lines and tumor xenograft mouse models. Then we evaluated the functions of AEBP1 on cell proliferation, migration, invasion, lipid reactive oxygen species (ROS), labile iron pool (LIP) and free iron, lipid peroxidation, and expression levels of ferroptosis-related genes.

**Results:**

AEBP1 was highly expressed in oral cancer cells and tissues. AEBP1 silencing inhibited oral cancer cell proliferation, migration, and invasion after SSZ treatment. SSZ-induced ferroptosis is due to enhanced ROS level, free iron, and lipid peroxidation, which were distinctly increased by AEBP1 silencing. Meanwhile, AEBP1 silencing enhanced the effects of SSZ on levels of LIP and Fe^2+^, lipid peroxidation, as well as the expression levels of ferroptosis-related genes in the tumor xenograft mouse models. Importantly, AEBP1 silencing suppressed tumor growth in vivo. Furthermore, silencing of AEBP1 might activate the JNK/ P38 /ERK pathway.

**Conclusion:**

This research suggested that silencing of AEBP1 predisposes cisplatin-resistant oral cancer cells to ferroptosis via the JNK/p38 /ERK pathway.

**Supplementary Information:**

The online version contains supplementary material available at 10.1186/s12903-022-02503-9.

## Background

Oral cancer is a complex disease with a high fatality rate, and is primarily due to excessive smoking, alcohol and betel nut intake, and human papillomavirus (HPV) infections [1]. It usually manifests as oral ulcer lesions, exogenous, proliferative, or papilloma lesions, cystic lesions, and oral melanoma [2]. Surgery is the primary modality of treatment for oral cancer, but the probability rate has not improved [2]. Chemotherapy is the commonly used adjuvant treatment, including 5-fluorouracil (5-FU) and hydroxyurea, taxane, platinum drugs, and cetuximab [3]. Nevertheless, tumors display differing degrees of resistance due to the extensive and perpetual use of chemotherapeutics, leading to poor prognosis and chemotherapy failure [4, 5].

Recent research has reported that ferroptosis may be a new therapeutic method of cancer treatment [6, 7]. Ferroptosis inducers mainly including erastin, sulfasalazine (SSZ), and glutamate, combined with radiotherapy and chemotherapy will further decrease toxic side effects and optimize the treatment effect [8]. Furthermore, glutathione peroxidase 4 (GPX4) is a key regulator of ferroptosis, its silencing leads to selective persister cell ferroptosis in vitro and prevents tumor recurrence in mice [9]. It has been demonstrated that ferroptosis can enhance sensitivity of cancer cells to chemotherapeutic drugs in small cell lung cancer, hepatocellular carcinoma, renal cell carcinoma, and pancreatic carcinoma, thereby inhibiting cancer progress [10–14]. SSZ effectively enhances the intracellular anti-cancer activity of cisplatin by targeting xc(−) transporter in colorectal cancer cells [15]. Mitochondrial pyruvate carrier 1 regulates sensitivity to ferroptosis via epithelial-mesenchymal transition in drug-tolerant persister head and neck cancer cells [16]. However, few studies have reported that SSZ-induced ferroptosis can enhance sensitivity to cisplatin in oral cancer.

Adipocyte enhancer-binding protein 1 (AEBP1) is a transcriptional inhibitor involved in the regulation of key biological processes, such as adipogenesis, and inflammation [17]. Furthermore, AEBP1 is a potential oncogene, and its overexpression is related to the development and progression of tumors, such as stomach cancer, colorectal cancer, glioblastoma, and bladder cancer [17]. For example, AEBP1 is highly expressed and promotes cell proliferation in primary glioblastomas [18]. High expression of AEBP1 is related to bladder cancer stage and tumor patients’ prognosis [19]. Up-regulation of AEBP1 contributes to tumor angiogenesis in colorectal cancer [20]. However, the expression of AEBP1 and the role of AEBP1 silencing on ferroptosis in oral cancer are unclear.

In this research, we demonstrated the role of AEBP1 silencing in promoting ferroptosis via the JNK/P38/ERK pathway in cisplatin-resistant oral cancer cells. The findings suggested that AEBP1 could be effective in the treatment of cisplatin-resistant oral cancer.

## Methods

### Cell culture

The human oral cells line (HOK) and oral cancer cell lines (CAL27 and SCC15) were all obtained from the American Type Culture Collection (Manassas, VA, USA). The cell lines were cultured in DMEM (Sigma Aldrich, St. Louis, MO, USA) supplemented with 10% FBS, 1% streptomycin, and 1% penicillin at 37 °C in 5% CO_2_, and 95% humidity. Cells were randomly divided into NT (non-SSZ treatment) and SSZ groups, respectively. Cells of the SSZ group were treated with SSZ (0.1, 0.5, and 1 mM, respectively) for 8 h. The cells of the NT group were treated with normal medium.

### Establishment of cisplatin-resistant cell lines and transfection

The cisplatin-resistant cell line (CAR) was established by clonal selection of CAL27 with 10–100 μM cisplatin treatment for 10 cycles followed by another generation of recovery, which are based on the research conducted by Chang et al [21]

### Transfection

The construction of shRNA targeting AEBP1 (sh-AEBP1) and non-targeting control (sh-NC) were obtained from GeneChem (Shanghai, China) (Table 1). sh-AEBP1 and sh-NC were transfected with oral cancer cells and using Lipofectamine 3000 (Invitrogen, Carlsbad, CA, USA) and were incubated for 48 h. The transfection efficiency of AEBP1 was determined by qRT-PCR. Then the transfected cells were collected for subsequent experiments.

**Table 1:** Sequences of AEBP1 knockdown and control shRNA in this study

Gene	Forward	Reverse
sh-AEBP1–1	5′-CACCGCCAGACATGGGTGATGTACACGAATGTACATCACCCATGTCTGGC-3’	5′-AAAAGCCAGACATGGGTGATGTACATTCGTGTACATCACCCATGTCTGGC-3’
sh-AEBP1–2	5′-CACCGCTATGAGGAAATGACCTTTCCGAAGAAAGGTCATTTCCTCATAGC-3’	5′-AAAAGCTATGAGGAAATGACCTTTCTTCGGAAAGGTCATTTCCTCATAGC-3’
sh-NC	5′-CCGGTTACGCGTAGCGTAATACGCTCGAGCGTATTACGCTACGCGTAATTTTTG-3’	5′-AATTCAAAAATTACGCGTAGCGTAATACGCTCGAGCGTATTACGCTACGCGTAA-3’

### Western blot assay

Protein was extracted using cell lysis buffer (Cell Signaling Technology, Danvers, MA, USA). The protein samples were resolved by 10% SDS-PAGE and were transferred to the PVDF membrane. Next, the membranes were blocked with 5% skim milk at 25 °C for 1 h, then were incubated with the primary antibodies AEBP1 (1:1000, ab168355, Abcam), JNK1/2 (1:1000, ab112501, Abcam), p-JNK1/2 (1:1000, ab4821, Abcam), p38 (ab170099, Abcam), p-p38 (1:1000, ab178867, Abcam) and GAPDH (ab9485, Abcam) overnight at 4 °C. Subsequently, the membranes were incubated with horseradish peroxidase (HRP)-conjugated secondary antibody for 1 h at 25 °C. Blots were detected by enhanced chemiluminescence reagent and western blotting reagents. GAPDH was employed as a protein loading control.

### Reverse transcription-quantitative PCR (RT-qPCR)

Total RNA was extracted using an RNA extraction kit (Sigma-Aldrich). RNA was reversed transcribed into cDNA using a Reverse Transcriptase Kit (Sigma-Aldrich) and RT-PCR was performed using the SYBR® Green Quantitative RT-PCR Kit (Sigma-Aldrich) according to the instructions of manufacturer. The Mastercycler ep realplex detection system (Eppendorf, Hamburg, Germany) was used for RT-qPCR assay. The sequences of primers are shown in Table 2.

**Table 2:** Primers for qRT-PCR in this study

Gene	Forward	Reverse
AEBP1	5′-AGACCACGCCATCTTCCG-3′	5′-CCTTGTTGTTCTCCCACTCG-3′
FTH1	5′-CGCCAGAACTACCACCAG-3′	5′-TTCAAAGCCACATCATCG-3′
GPX4	5′-GAAGCAGGAGCCAGGGAGT-3′	5′-ACGCAGCCGTTCTTGTCG-3′
COX2	5′-TGGAGCACCATTCTCCTTGAAAGGACTTAT-3’	5′-GACTGTTTTAATGAGCTCTGGATCTGGAAC-3’
SLC7A11	5′-TGCTGGGCTGATTTTATCTTCG-3’	5′-GAAAGGGCAACCATGAAGAGG-3’
GAPDH	5′-GAATTCATGTTTGAGACCTTCAA-3’	5′-CCGGATCCATCTCTTGCTCGAAGTCCA −3’

### 3-[4,5-dimethyl-2-thiazolyl]-2,5 diphenyl-2H-tetrazolium bromide (MTT) assay

Cell viability was measured using the MTT kit (Sigma-Aldrich). The cells were cultured into 96-well plates for 24 h. Then, 20 μl of MTT (2.5 mg/ml) was added to the wells and maintained at 37 °C for 4–6 h. Afterward, the formazan crystals were dissolved by dimethyl sulfoxide (DMSO) after the medium was removed. Finally, the absorbance was measured at 450 nm using a SpectraMax M2 microplate reader (Molecular Devices, Sunnyvale, CA).

### 5-Ethynyl-20-deoxyuridine (EdU) cell proliferation assay

According to the instructions of the manufacturer, the proliferation of oral cancer cells was detected by the EdU kit (Ribobio, Guangzhou, China). The cell nuclei were counter stained with 1 mg/ml DAPI for 5 min. Finally, the images were acquired by the fluorescence microscope (Leica, Germany), and the EdU positive cell ratio was calculated.

### Transwell migration and invasion assay

The migration and invasion of oral cancer cells were measured by transwell (8 μm pore, Corning, Inc.). For cell migration assay, cells were cultured in serum-free RPMI-1640 overnight and were added into the upper chambers (3 × 10^4^ cells). And the lower chamber was added into the medium containing 10% FBS. After incubating for 24 h at 37 °C, cells in the lower chamber were fixed with 4% paraformaldehyde for 30 min and stained with 0.1% crystal violet for 10 min at 25 °C. Then the migrated cells were counted by the light microscope. For cell invasion assay, the upper surface of the transwell chambers was pre-coated with Matrigel (BD Biosciences, Sparks, USA) for 5 h at 37 °C. Other operations of invasion are consistent with cell migration assay.

### Measurement of ROS production, GSH level, and lipid peroxidation

Oral cancer cells were treated with 1 mM SSZ for 8 h. Then the cellular ROS and lipid ROS were detected using a ROS detection kit (Sigma-Aldrich) according to the instructions of the manufacturer. Cellular glutathione (GSH) levels were measured using a GSH colorimetric detection kit (Sigma-Aldrich). Cellular lipid peroxidation was also evaluated by determining the malondialdehyde (MDA) concentration using a lipid peroxidation detection kit (Sigma-Aldrich).

### Labile iron pool (LIP) and ferrous iron assays

The total cellular LIP was detected based on the calcein-acetoxymethyl ester method. Cells were treated with 2 μM calcein acetoxymethyl ester (Sigma-Aldrich) at 37 °C for 30 min, then washed with hanks balanced salt solution. The final concentration of 100 μM deferoxamine mesylate is used to remove the iron in calcein. Then, the cells were incubated with or without deferoxamine for 1 h at 37 °C. Fluorescence was measured at 485 nm excitation and 535 nm emissions using the fluorescence plate reader (BioTek, Winooski, VT, USA). The fluorescence change was used as an indirect measurement of LIP after the addition of deferoxamine. The Fe^2+^ level in cells or mitochondria was detected using an iron detection kit (Sigma-Aldrich). The tissues or the cells collected were immediately homogenized with phosphate-buffered saline (PBS). After centrifugation, the supernatant was assayed for iron concentration using the Kit according to the manufacturer’s instructions.

### Tumor xenograft model

The athymic BALB/c male nude mice (5 weeks old) were obtained from Esebio (Shanghai, China). The CAR cells (40 μl, 0.5 × 10^6^ cells) with sh-AEBP1 and sh-NC were subcutaneously injected into the bilateral flank of nude mice. To explore the role of AEBP1 on the cisplatin-resistance of oral cancer cells, therefore, some mice were divided into sh-NC, sh-NC + cisplatin, sh-AEBP1, and sh-AEBP1 + cisplatin groups. From the day when gross nodules were found in tumor implants, the mice of sh-NC + cisplatin and sh-AEBP1 + cisplatin groups were received cisplatin (25 mg/kg) by daily intraperitoneal injection. Furthermore, to further explore the interaction between AEBP1 and SSZ in cisplatin-resistant oral cancer cells, other mice were divided into sh-NC, sh-NC + SSZ, sh-AEBP1, and sh-AEBP1 + SSZ groups. The mice of sh-NC + SSZ and sh-AEBP1 + SSZ groups were intraperitoneal injected SSZ (250 mg/kg) daily. They were euthanized after 28 days. The tumor volume and weight, and body weight were measured every 7 days. The tumor volume was calculated as (length × width^2^)/2. The animal research procedures were carried out in accordance with the the China Animal Welfare Legislation and were approved by the Ethics Committee of Maternal and Child Health Hospital of Hubei Province.. The study is reported in accordance with ARRIVE guidelines.

### Statistical analysis

Data were presented as mean ± standard deviation. All statistical tests were analyzed using Student’s t-test, one-way ANOVA, and two-way ANOVA followed by Tukey’s post hoc tests by SPSS 22.0 (IBM, Armonk, NY, USA). *P* < 0.05 was considered to be statistically significant.

## Result

### AEBP1 is up-regulated in oral cancer cells

**Fig. 1:**
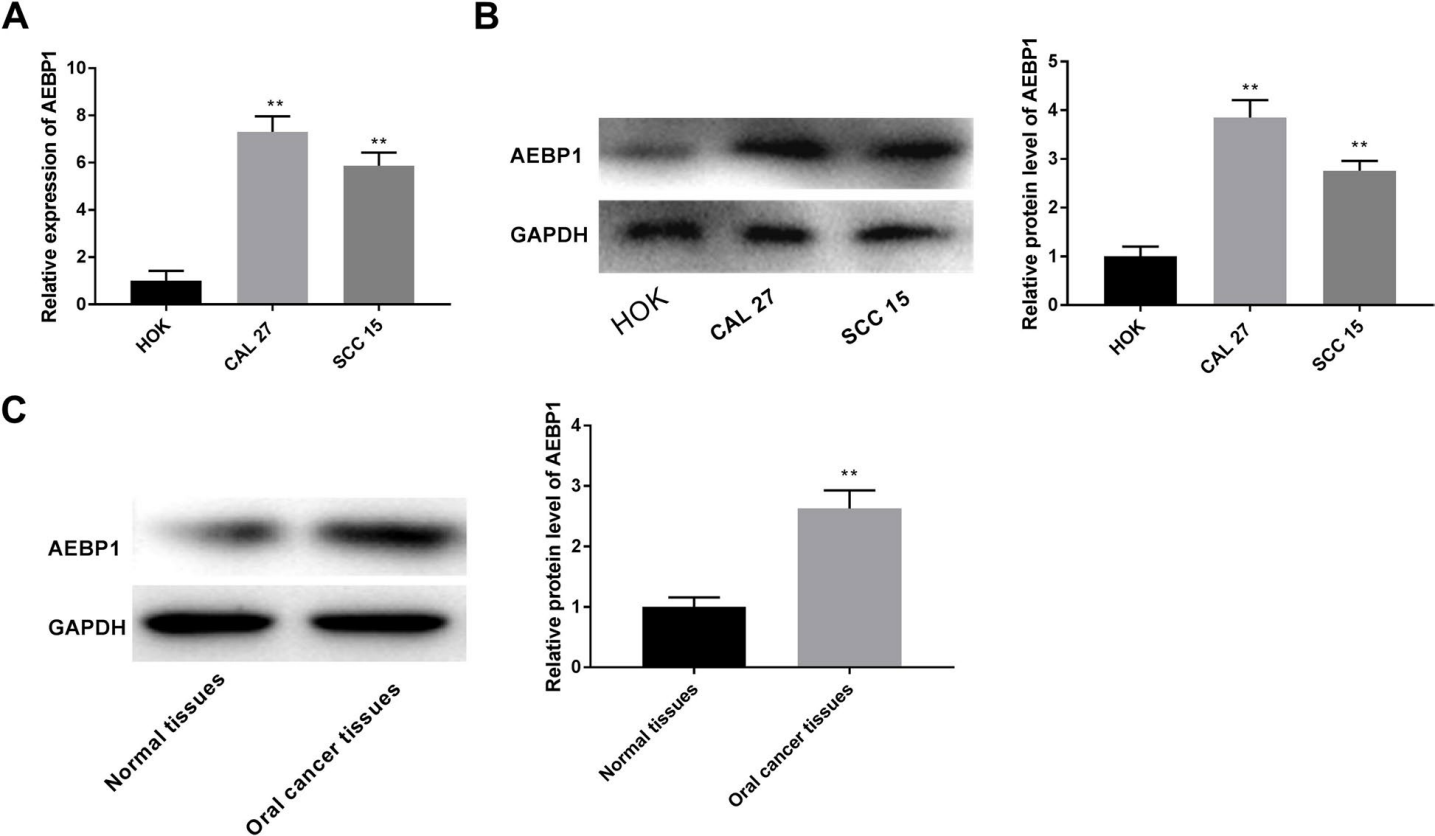
AEBP1 is up-regulated in oral cancer cells. **A**, **B** mRNA and protein expression of AEBP1 was detected by RT-qPCR and western blot. **P < 0.01 versus HOK cells

The expression of AEBP1 was detected in different cells by RT-qPCR and western blot. The data showed that AEBP1 was highly expressed in CAL27 and SCC15 cells compared with the HOK cells (Fig. 1A-B, *P* < 0.01). In addition, AEBP1 was up-regulation in oral cancer tissues compared with normal tissues (Fig. 1C, *P* < 0.01). CAL27 cells were selected for subsequent experiments due to the relatively high expression level of AEBP1.

### AEBP1 silencing inhibits cell proliferation, migration, and invasion of CAL27 and CAR cells

**Fig. 2:**
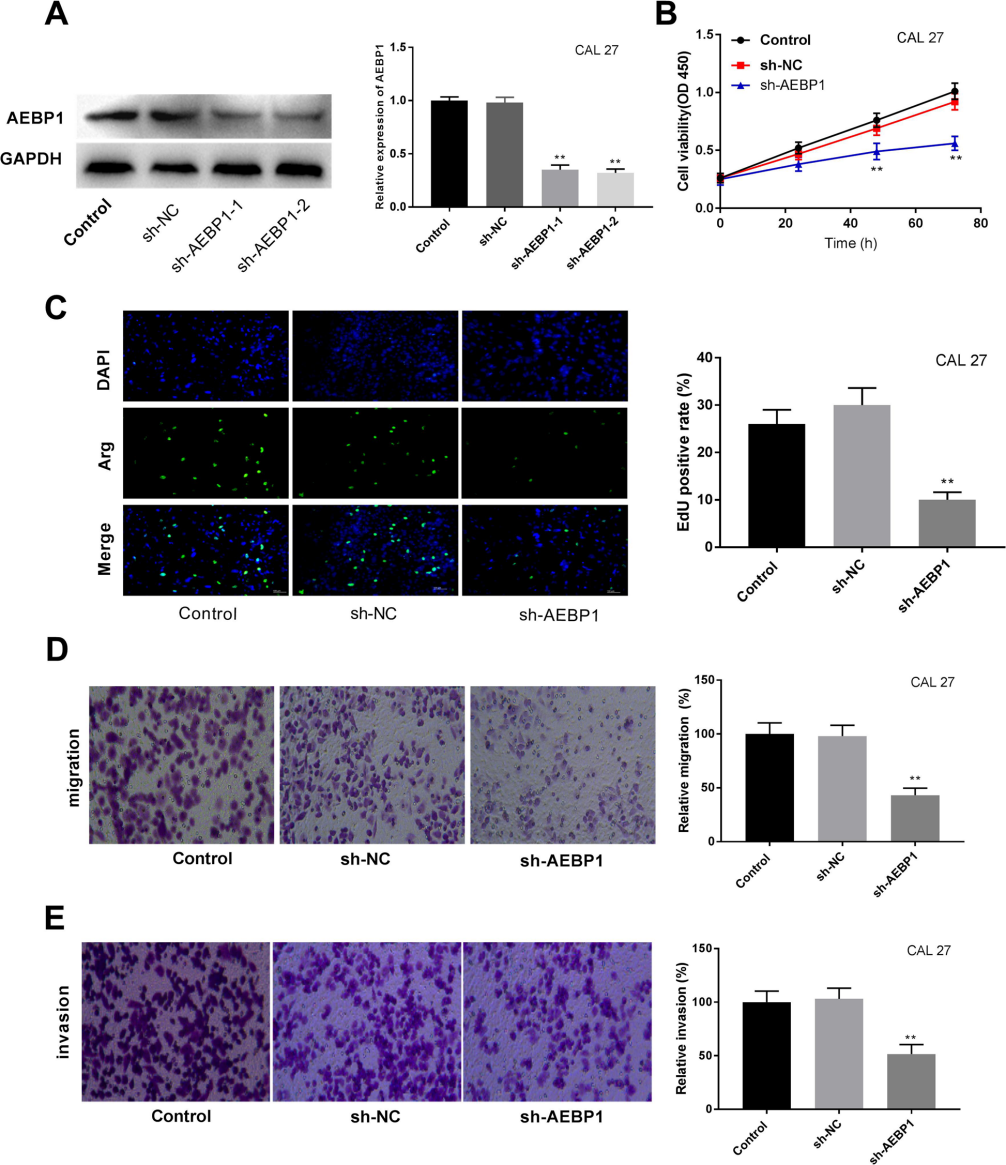
AEBP1 silencing inhibits cell proliferation, migration, and invasion of CAL27 cells. **A** The AEBP1 expression was detected by RT-qPCR in CAL27 cells. **B** Cell viability was measured by MTT assay in CAL27 cells. **C** The proliferation of CAL27 cells was detected by the EdU detection kit. **D**, **E** Cell migration and invasion were determined by transwell assay. **P < 0.01 versus sh-NC

**Fig. 3:**
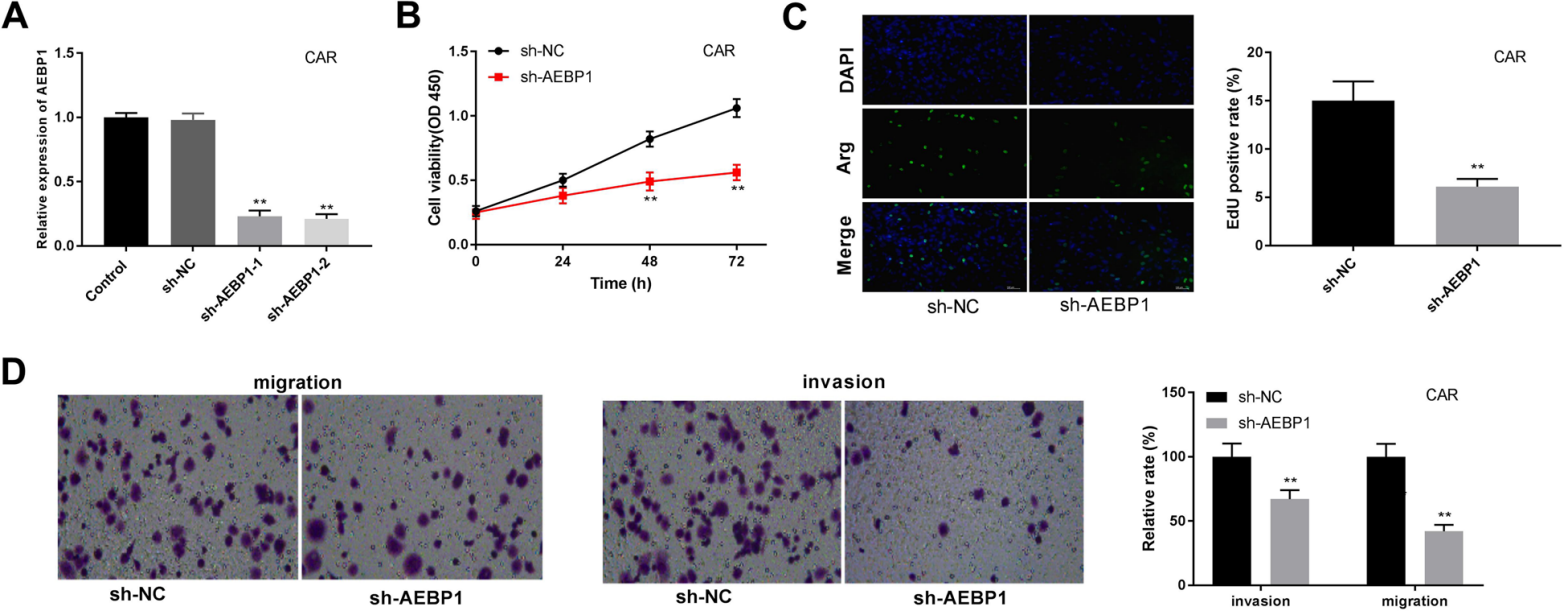
AEBP1 silencing inhibits cell proliferation, migration, and invasion of CAR cells. **A** The AEBP1 expression was detected by RT-qPCR in CAR cells. **B** Cell viability was measured by MTT assay in CAR cells. **C** The proliferation of CAR cells was detected by the EdU detection kit. **D**, **E** Cell migration and invasion were determined by transwell assay. **P < 0.01 versus sh-NC

To assess the function of AEBP1 on oral cancer cells, AEBP1 was knocked down by shRNA transfection in CAL27 cells. The expression of AEBP1 was decreased after AEBP1 silencing (Fig. 2A, *P* < 0.01). AEBP1 silencing decreased the viability of CAL27 cells (Fig. 2B, *P* < 0.01). The EdU-positive cells were decreased by sh-AEBP1 (Fig. 2C, *P* < 0.01). Furthermore, as shown in Fig. 2D-E, sh-AEBP1 inhibited migration and invasion of CAL27 cells (*P* < 0.01). Meanwhile, similar results were shown in CAR cells. The silencing of AEBP1 inhibited the viability, proliferation, migration, and invasion of CAR cells (Fig. 3A-D, *P* < 0.01). The results indicated that AEBP1 silencing suppressed the proliferation, migration, and invasion of oral cancer cells, thereby contributing to suppressing the progress of oral cancer cells.

### AEBP1 silencing sensitizes oral cancer cells to SSZ treatment in vitro

**Fig. 4:**
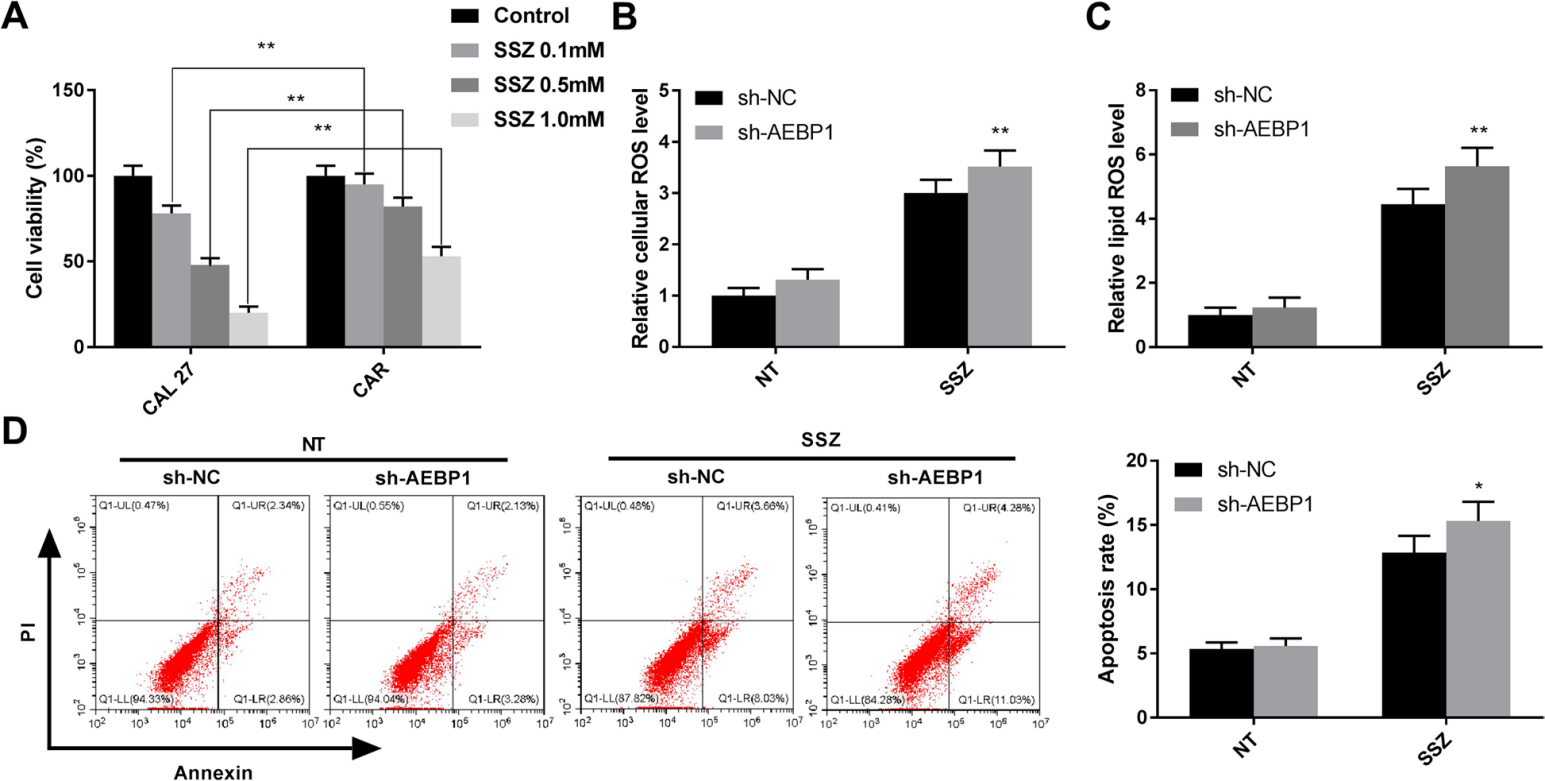
AEBP1 silencing sensitizes oral cancer cells to SSZ treatment in vitro. **A** Cell viability after SSZ treatment in CAL27 cells and CAR cells. **P < 0.01 versus the CAL27 cells. **B**, **C** Cellular ROS level and lipid ROS level were detected by corresponding detection kit. **P < 0.01 versus sh-NC

The effect of AEBP1 silencing on SSZ-induced ferroptosis was explored in oral cancer cells. The viability of CAL27 cells was reduced in a dose-dependent manner of SSZ (Fig. 4A). The CAR cells were comparatively less sensitive to SSZ treatment compared with CAL27 cells (Fig. 4A, *P* < 0.01). Furthermore, one of the key factors regulating ferroptosis is the production of ROS [22]. Knockdown of AEBP1 had no significant effects on cellular ROS and lipid ROS compared with sh-NCs. However, after SSZ treatment, the data showed that AEBP1 silencin*g* increased the cellular ROS level in CAL27 cells (Fig. 4B, *P* < 0.01). The silencing of AEBP1 also significantly enhanced the effects of SSZ on lipid ROS level in CAL27 cells (Fig. 4C, *P* < 0.01). In addition, AEBP1 silencing promoted cell apoptosis after SSZ treatment (Fig. 4D, *P* < 0.05). The data showed that inhibition of AEBP1 promotes ferroptosis of oral cancer cells.

### AEBP1 silencing promotes ferroptosis of CAR cells in vitro

**Fig. 5:**
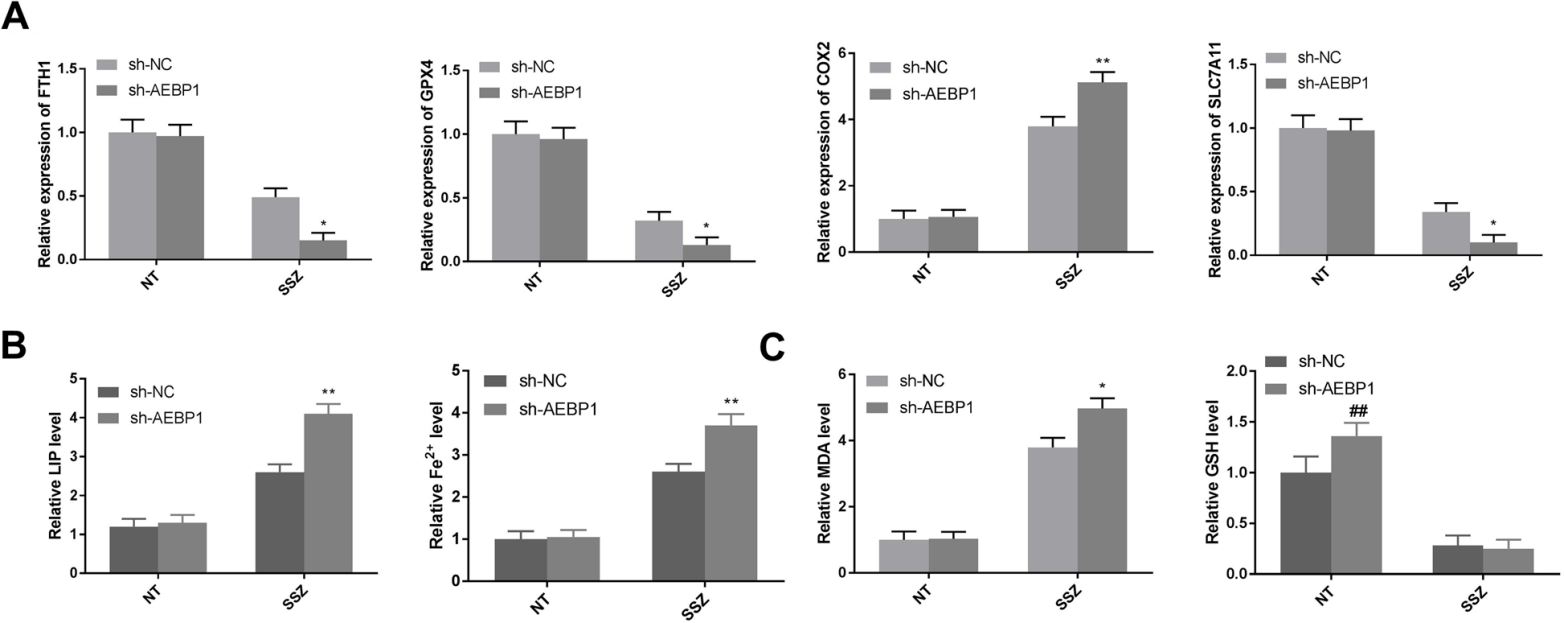
AEBP1 silencing promotes ferroptosis of CAR cells in vitro. **A** The expression levels of FTH1, GPX4, COX2, and SLC7A11 were detected by RT-qPCR. **B**, **C** MDA, GSH, LIP and Fe^2+^ levels were detected in sh-NC, sh-NC + SSZ, sh-AEBP1, and sh-AEBP1 + SSZ groups. *P < 0.05, **P < 0.01 versus sh-NC + SSZ. ^##^*P* < 0.01 versus sh-NC

Some research has reported that ferritin heavy chain 1 (FTH1) [23], cystine transporter SLC7A11 [24], and GPX4 [25] can inhibit ferroptosis, while cyclooxygenase-2 (COX2) [26, 27] can promote ferroptosis. To investigate whether AEBP1 can regulate ferroptosis of cisplatin-resistant oral cancer, therefore, the levels of free iron, lipid peroxidation, and ferroptosis-related genes in CAR cells were detected. As shown in Fig. 5A, AEBP1 silencing decreased the levels of FTH1, GPX4, and SLC7A11 (*P* < 0.05), while COX2 level was increased after SSZ treatment (*P* < 0.01). The LIP level and Fe^2+^ level were distinctly increased after SSZ treatment, and AEBP1 silencing enhanced the effects of SSZ on LIP and Fe^2+^ levels (Fig. 5B, *P* < 0.01). In addition, SSZ treatment increased MDA level, and AEBP1 silencing enhanced the effect of SSZ treatment on MDA level (Fig. 5C, *P* < 0.05). GSH level was reduced by SSZ treatment, while the change in GSH level did not differ distinctly between the sh-NC + SSZ group and sh-AEBP1 + SSZ group. The results suggested that AEBP1 silencing might promote ferroptosis through regulating ferroptosis-related genes, increasing iron accumulation, and lipid peroxidation, thereby sensitizing CAR cells to SSZ treatment in vitro.

### AEBP1 interacts with the JNK/ P38 ERK pathway

**Fig. 6:**
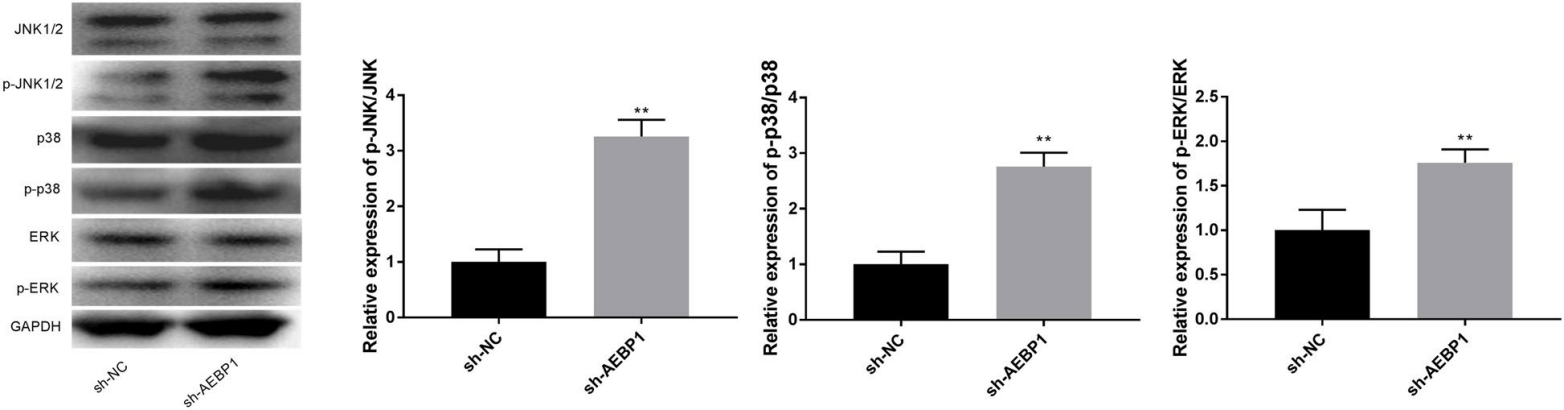
AEBP1 interacts with the JNK/ P38/ERK pathway. **A** The levels of p38, p-P38, JNK1/2, p-JNK1/2, ERK, and p-ERK were detected by western blot. **P < 0.01 versus sh-NC

In order to clarify the regulatory mechanism of AEBP1 on ferroptosis, MAPK pathway-related genes were detected. The data showed that the levels of p-P38, p-JNK1/2, and p-ERK were increased after AEBP1 silencing (Fig. 6, *P* < 0.01). The results suggested that silencing of AEBP1 might interact with the JNK / P38 / ERK pathway, thereby suppressing tumor progress.

### AEBP1 silencing sensitizes CAR cells to SSZ treatment in vivo

**Fig. 7:**
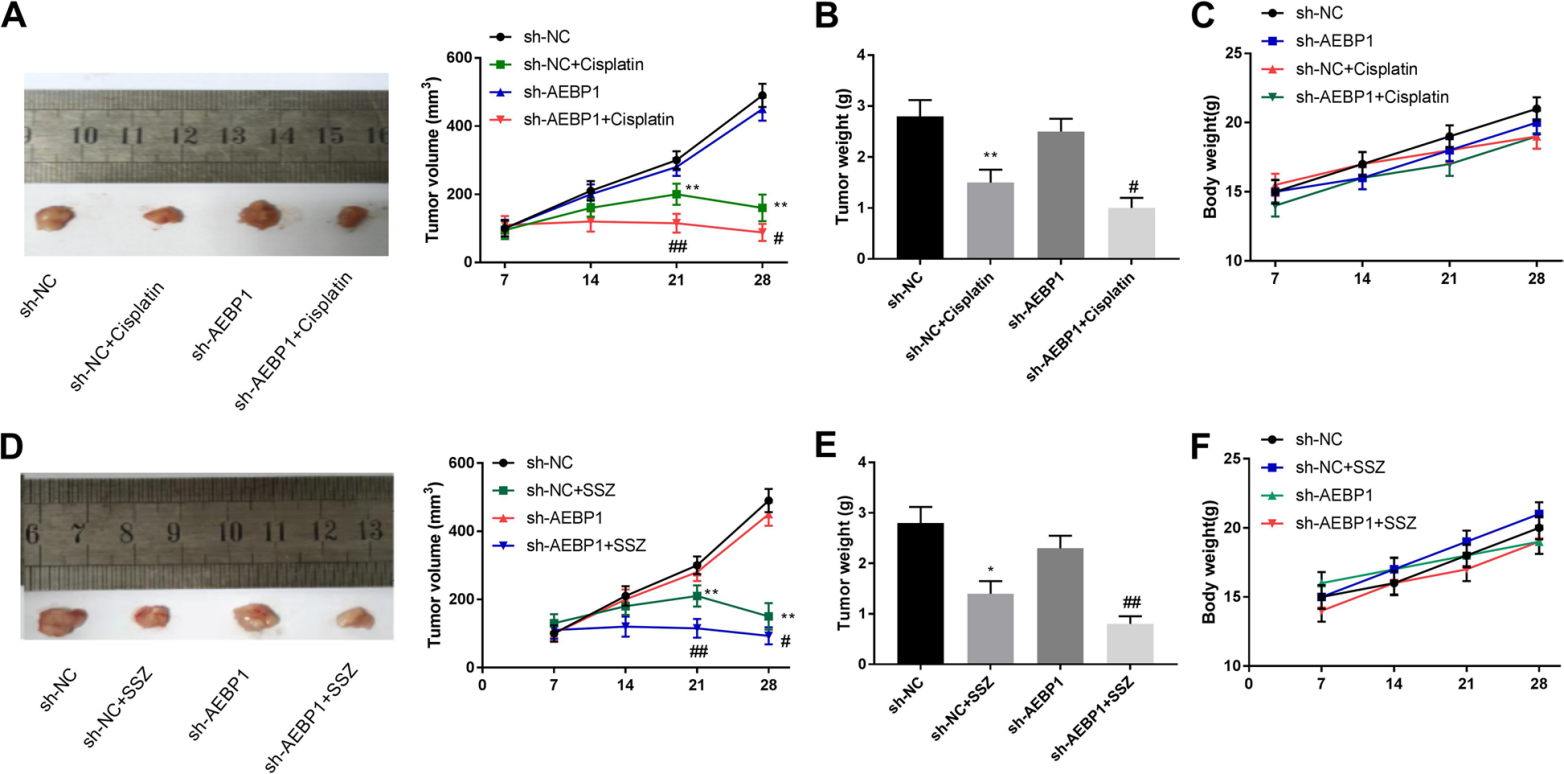
AEBP1 silencing sensitizes CAR cells to SSZ treatment in vivo. **A**-**C**. Tumor volume, tumor weight and body weight in sh-NC, sh-NC + cisplatin, sh-AEBP1 and sh-AEBP1 + cisplatin groups. **P < 0.01 versus sh-NC. ^#^*P* < 0.05, ^##^*P* < 0.01 versus sh-NC + cisplatin. **D**-**F** Tumor volume, tumor weight and body weight in sh-NC, sh-NC + SSZ, sh-AEBP1 and sh-AEBP1 + SSZ groups. *P < 0.05, **P < 0.01 versus sh-NC. ^#^*P* < 0.05, ^##^*P* < 0.01 versus sh-NC + SSZ

The role of AEBP1 silencing on cisplatin-resistant tumors in vivo was evaluated. As shown in Fig. 7A-B, cisplatin treatment decreased the tumor volume and tumor weight (*P* < 0.01), and the silencing of AEBP1 significantly enhanced the inhibition effects of cisplatin on tumor growth. Similarly, SSZ treatment significantly suppressed the volume and weight of tumor compared with the sh-NC group, which was more significant in the sh-AEBP1 + SSZ group than in the sh-NC + SSZ group (Fig. 7D-E, *P* < 0.01). Body weight did not change significantly (Fig. 7C-F). The results suggested that AEBP1 promotes tumor growth and AEBP1 silencing contributes to suppressing tumorigenesis.

### AEBP1 silencing promotes ferroptosis of CAR cells in vivo

**Fig. 8:**
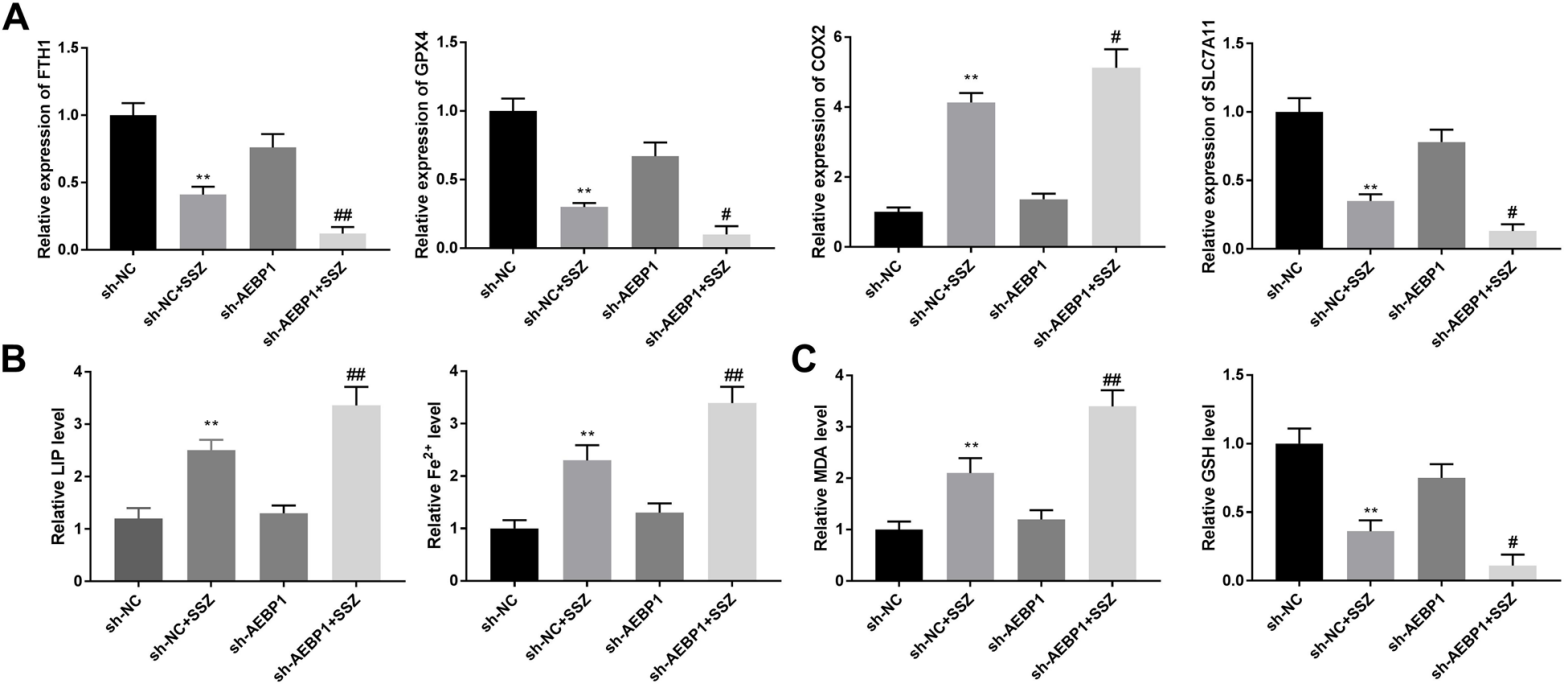
AEBP1 silencing promotes ferroptosis of CAR cells in vivo. **A** The expression levels of FTH1, GPX4, COX2, and SLC7A11 were detected by RT-qPCR. **B**, **C** The levels of LIP, Fe^2+^, MDA, and GSH were detected in sh-NC, sh-NC + SSZ, sh-AEBP1, and sh-AEBP1 + SSZ groups. **P < 0.01 versus sh-NC. ^#^*P* < 0.05, ^##^*P* < 0.01 versus sh-NC + SSZ

We evaluated whether AEBP1 was involved in ferroptosis regulation of CAR cells in vivo. Similar results were shown in mouse tumor xenograft models. As shown in Fig. 8A, the data showed that AEBP1 silencing significantly enhanced the effects of SSZ on expression levels of FTH1 (*P* < 0.01), GPX4 (*P* < 0.05), SLC7A11 (*P* < 0.05) and COX2 (*P* < 0.05). And AEBP1 silencing enhanced the effects of SSZ on LIP and Fe^2+^ levels (Fig. 8B, *P* < 0.01). Furthermore, as shown in Fig. 8C, AEBP1 silencing significantly enhanced the effects of SSZ on MDA level (*P* < 0.01) and GSH level (*P* < 0.05). The results indicated that inhibition of AEBP1 promotes ferroptosis through increasing lipid peroxidation, free iron accumulation, and regulating the expression of ferroptosis-related genes, thereby sensitizing CAR cells to SSZ treatment in vivo.

## Discussion

The mortality rate of oral cancer is close to 50%. Although there have been advancements in diagnostic techniques and therapy methods, the prognostic effect is indigent [28, 29]. Consequently, an effective chemotherapy mechanism is required to enhance the sensitivity of tumors to chemotherapeutic drugs. Ferroptosis is an iron-regulated cell death type and is caused by ROS accumulation and lipid peroxidation [30, 31]. Ferrous iron can selectively kill tumor cells by reacting with lipid peroxides to form cytotoxic lipid free radicals [32]. Cellular iron homeostasis is maintained by iron metabolism and the iron-dependent protein network to regulate the expression of iron-related proteins [33, 34]. Therefore, it is an effective strategy to treat cancer by enhancing the accumulation of ROS and free iron in cancer cells to promote ferroptosis. In this study, we demonstrated that AEBP1 is a critical regulator of ferroptosis, and AEBP1 silencing promotes ferroptosis by the JNK / p38 / ERK pathway in cisplatin-resistant oral cancer.

Currently, the function of AEBP1 in promoting carcinogenesis has been reported, and up-regulation of AEBP1 expression predisposes tumorigenesis in various cancer samples. For instance, AEBP1 silencing inhibits cell proliferation and increases apoptotic in primary glioblastomas [35]. Silencing of AEBP1 inhibits proliferation, migration, and tube formation of human umbilical vein endothelial cells [20]. AEBP1 silencing suppresses proliferation, invasion, migration, and metastasis of gastric cancer cells via the NF-κB pathway [36]. Based on previous research, we speculated that AEBP1 silencing contributes to inhibiting oral cancer progression. Consistent with previous research, in this research, the AEBP1 mRNA and protein expression were up-regulated in CAL27 and SCC15 cells. Furthermore, AEBP1 silencing inhibited proliferation, migration, and invasion in CAL27 cells. Taken together, these results indicated that AEBP1 might exert oncogenic effects in oral cancer progression, and AEBP1 silencing may suppress the progress of oral cancer by inhibiting proliferation, migration, and invasion.

Studies have reported that ferroptosis can suppress the proliferation of malignant tumor cells, such as head and neck cancer. CDGSH iron-sulfur domain-containing protein 2 (CISD2) gene silencing makes resistant head and neck tumor cells sensitive to SSZ-induced ferroptosis by increasing lipid ROS and Fe^2+^ levels [37]. The inhibition of GLRX5 enhances ROS level and lipid peroxidation, thereby predisposing to ferroptosis in therapy-resistant head and neck tumor cells [38]. Inhibition of cystine/glutamate antiporter overcomes the cisplatin resistance of head and neck cancer cells by inducing ferroptosis [39]. Based on previous studies, we speculated that AEBP1 silencing contributes to enhancing CAR cell sensitivity to SSZ-induced ferroptosis. In this research, cisplatin-resistant CAR cells were comparatively less sensitive to SSZ treatment. AEBP1 silencing promoted apoptosis after SSZ treatment. Furthermore, the suppression of AEBP1 increased cellular free iron, ROS level, and lipid peroxidation after SSZ treatment in vitro. Meanwhile, AEBP1 silencing decreased the levels of FTH1, GPX4, and SLC7A11, and increased COX2 level. The results demonstrated that silencing of AEBP1 can make oral cancer cells sensitive to SSZ-induced ferroptosis in vitro. Importantly, this conclusion was further supported by in vivo data. AEBP1 silencing enhanced the effects of SSZ on the levels of LIP, Fe^2+^, and lipid peroxidation, as well as regulated the expression of ferroptosis-related genes in vivo. Moreover, AEBP1 silencing suppressed tumor growth in the tumor xenograft mice models. The results demonstrated that AEBP1 silencing may promote ferroptosis of CAR cells in vitro and in vivo, thereby suppressing the progression of cisplatin-resistant oral cancer.

Mitogen-activated protein kinases (MAPK) are mammalian serine-threonine protein kinases including p38 MAPK, c-Jun NH2 terminal kinase (JNK), and extracellular signal-regulated kinase (ERK), which have key roles in human diseases development by stimulating proliferation, apoptosis, and inflammation [40]. For instance, methyl protodioscin with cathepsin S interact through the JNK/p38 pathway, thereby enhancing oral cancer cell sensitivity to chemotherapeutics [41]. Erastin-induced ferroptosis in human pancreatic islet-cell clusters is regulated via activation of JNK/P38/MAPK pathways [42]. High mobility group box 1 regulates ferroptosis by the RAS-JNK/p38 pathway in the pathogenesis of leukemia and chemotherapy resistance [43]. Therefore, based on previous research, we speculated that AEBP1 interacts with the JNK/p38/ERK pathway in cisplatin-resistant oral cancer cells. In this research, the expression of JNK, ERK, and p38 was up-regulated after AEBP1 silencing, suggesting AEBP1 silencing activated the JNK/p38/ERK pathway. Taken together, AEBP1 may exert effects by targeting the JNK/p38/ERK pathway in cisplatin-resistant oral cancer cells.

## Conclusions

In conclusion, the present study suggested that AEBP1 may be a novel regulator of ferroptosis by JNK/p38/ERK pathway and is also a potential therapeutic target in cisplatin-resistant oral cancer.

## Supplementary Information

**Additional file 1.**

## Data Availability

The datasets used and/or analysed during the current study are available from the corresponding author on reasonable request. The genes analyzed in the present study are available at https://www.ncbi.nlm.nih.gov/search/ (AEBP1, Gene ID:165).

## Abbreviations

AEBP1: adipocyte enhancer-binding protein 1
SSZ: sulfasalazine
ROS: reactive oxygen species
LIP: labile iron pool
HPV: human papillomavirus
