# Supplementary material for: Inhibition of AEBP1 predisposes cisplatin-resistant oral cancer cells to ferroptosis
Source: BMC Oral Health. 2022 Nov 9;22:478. doi: 10.1186/s12903-022-02503-9 (PMC9644533; doi:10.1186/s12903-022-02503-9)
Supplement: Supplementary file 1 — Additional file 1. [file 12903_2022_2503_MOESM1_ESM.pdf]

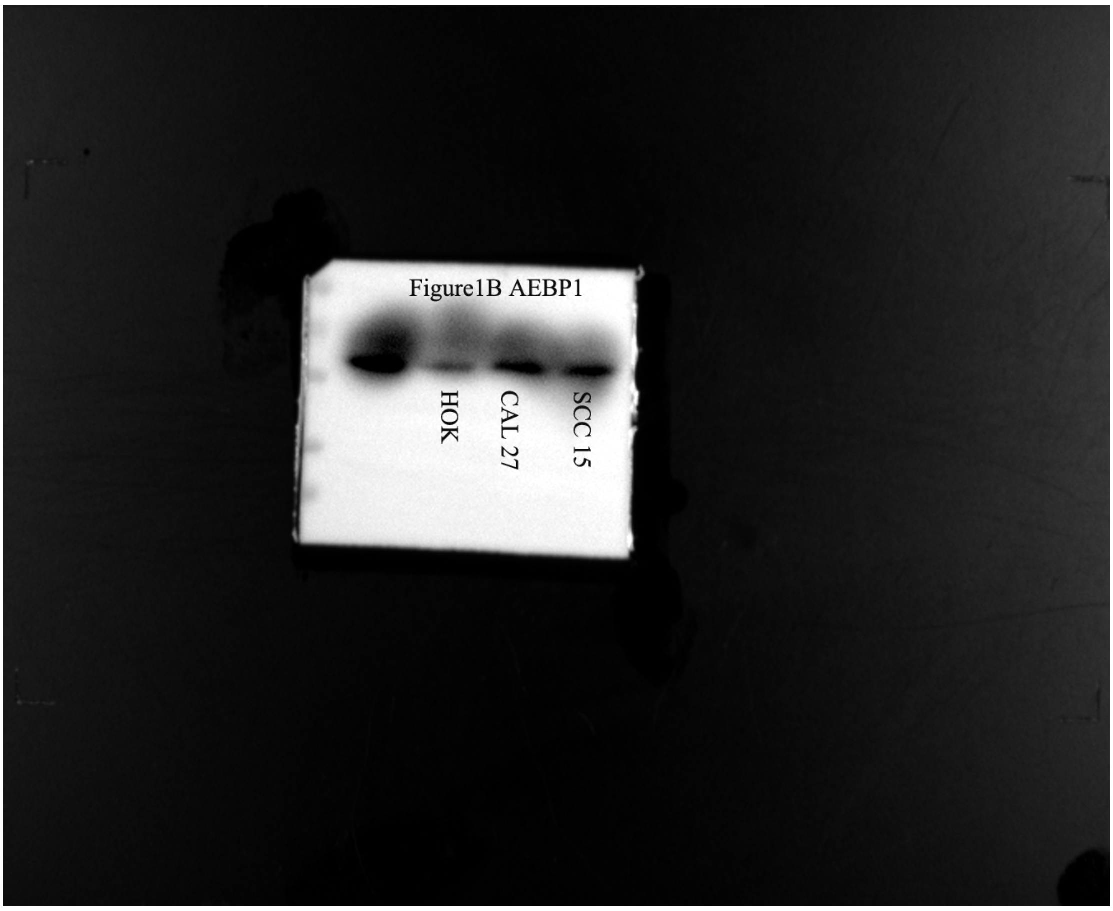
Figure1B AEBP1
HOK
CAL 27
SCC 15

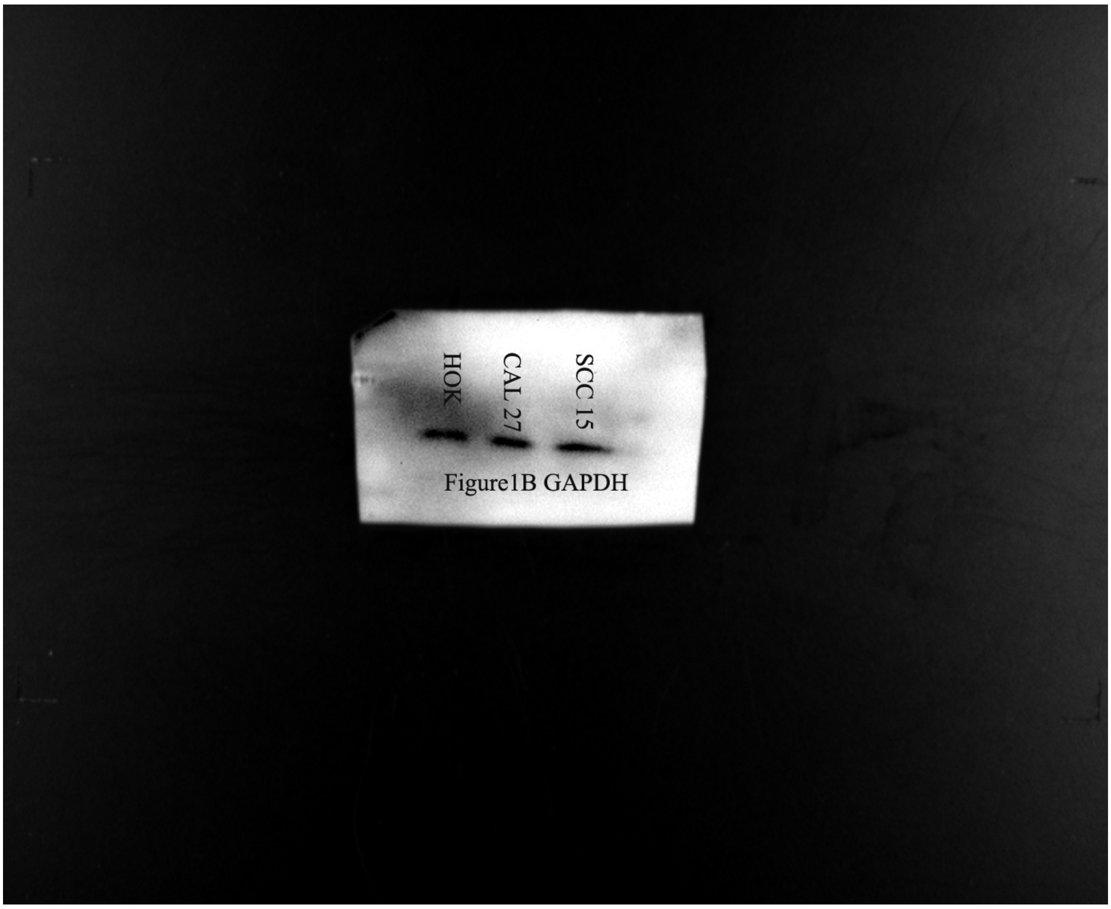
HOK
CAL 27
SCC 15
Figure1B GAPDH

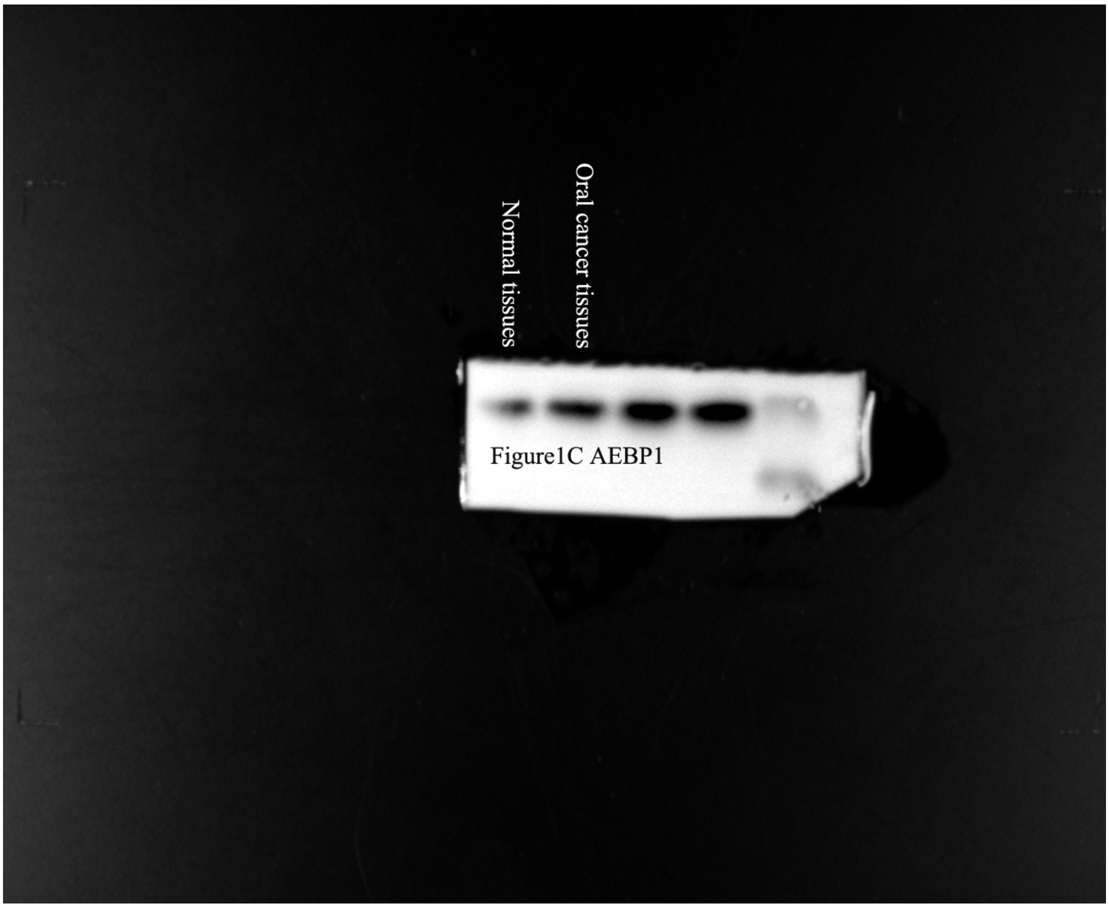

Figure1C AEBP1

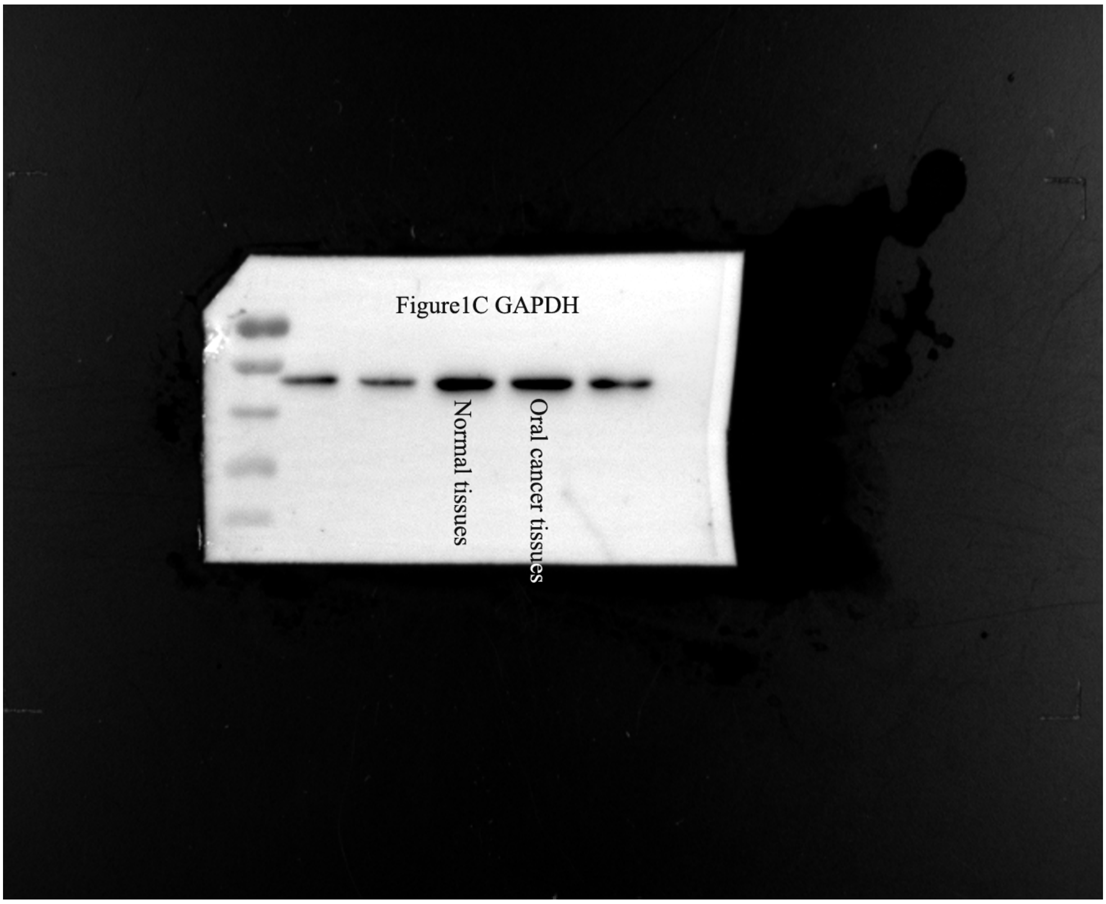
Figure1C GAPDH
Normal tissues
Oral cancer tissues

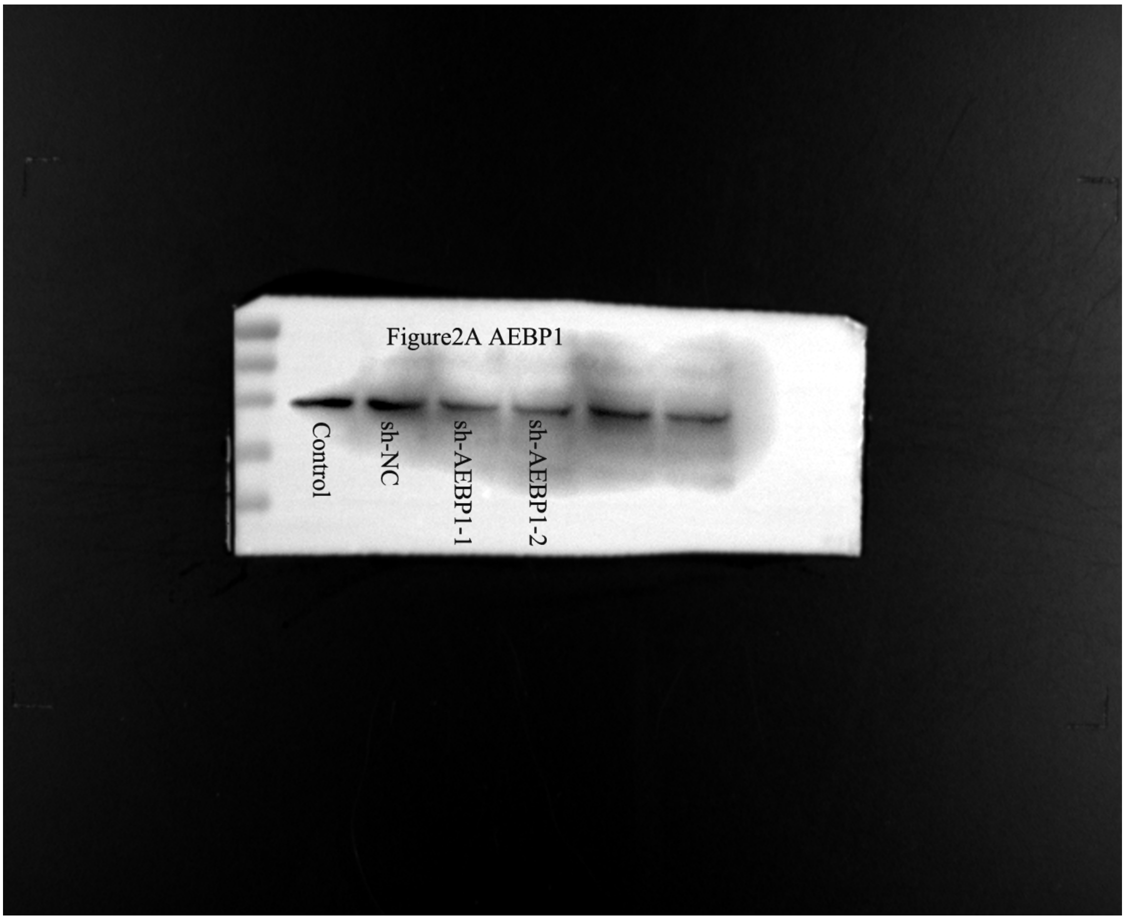
Figure2A AEBP1
Control
sh-NC
sh-AEBP1-1
sh-AEBP1-2

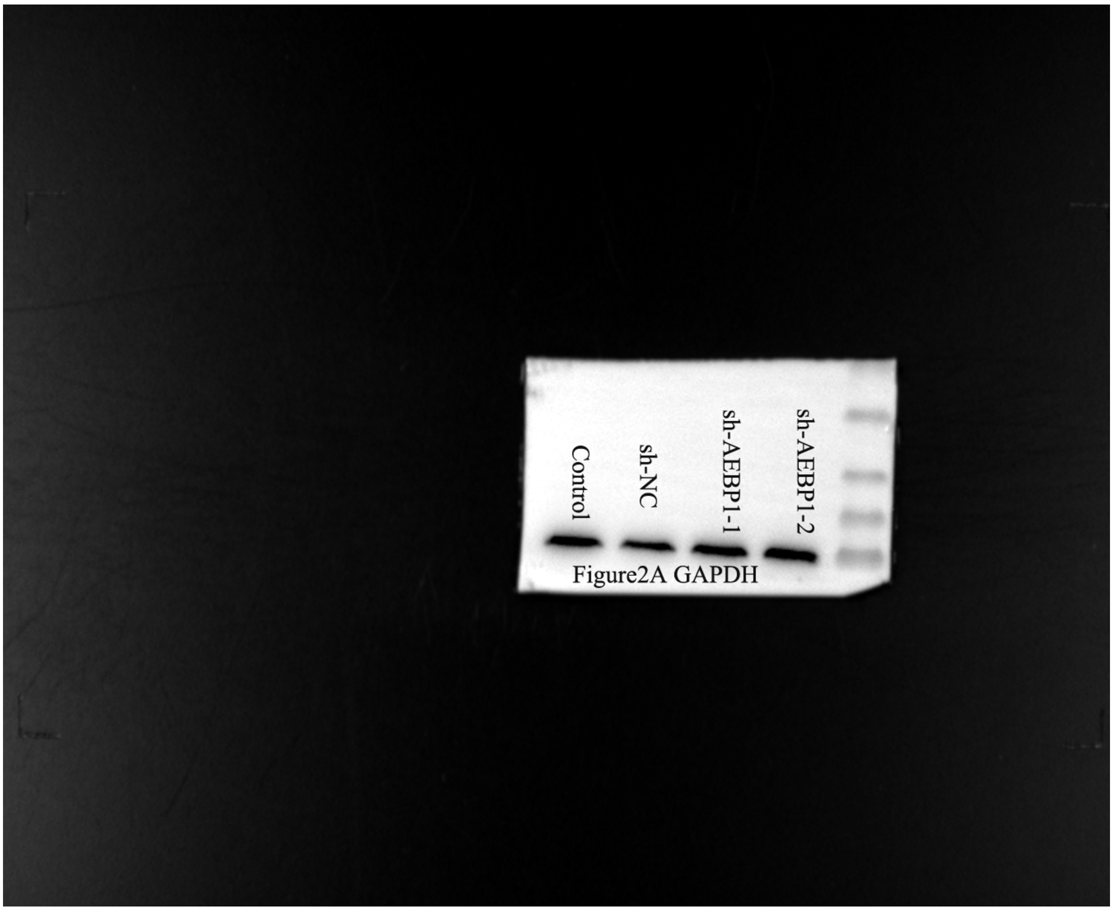
Control
sh-NC
sh-AEBP1-1
sh-AEBP1-2
Figure2A GAPDH

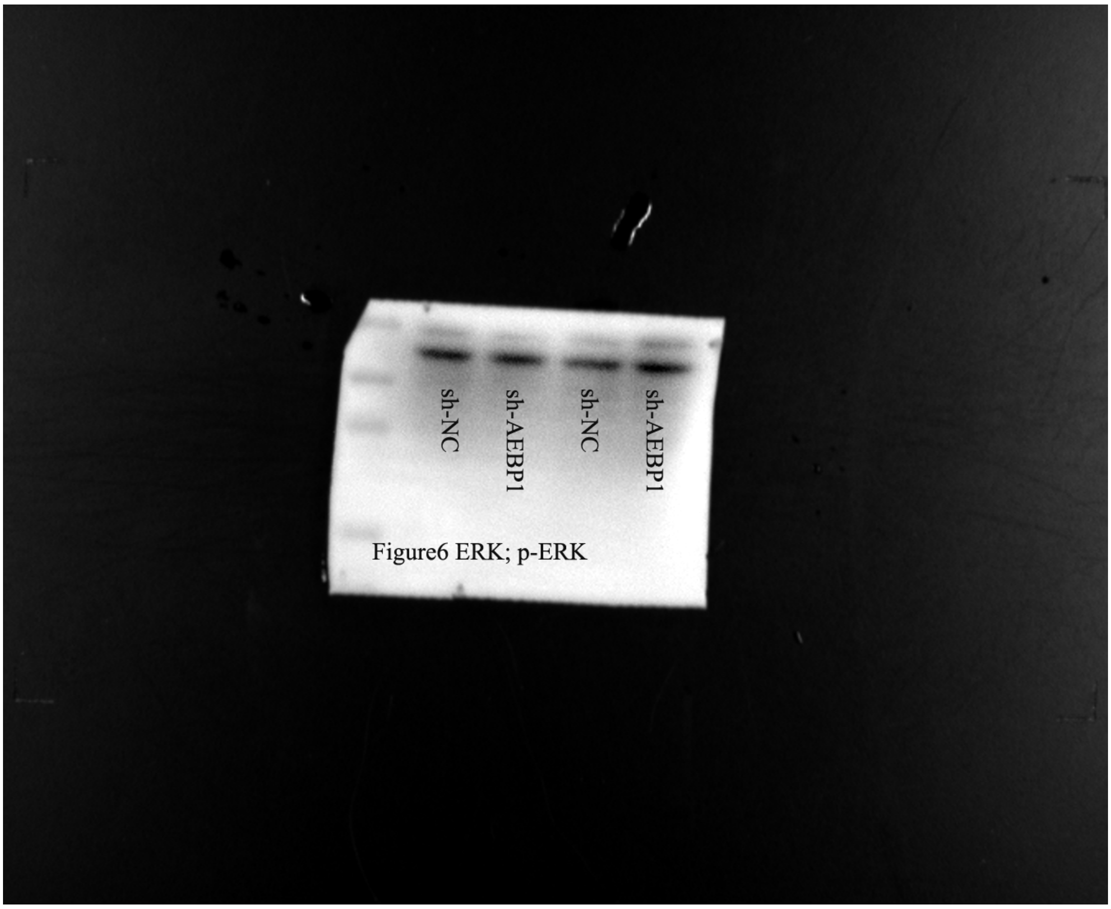
sh-NC
sh-AEBP1
sh-NC
sh-AEBP1
Figure6 ERK; p-ERK

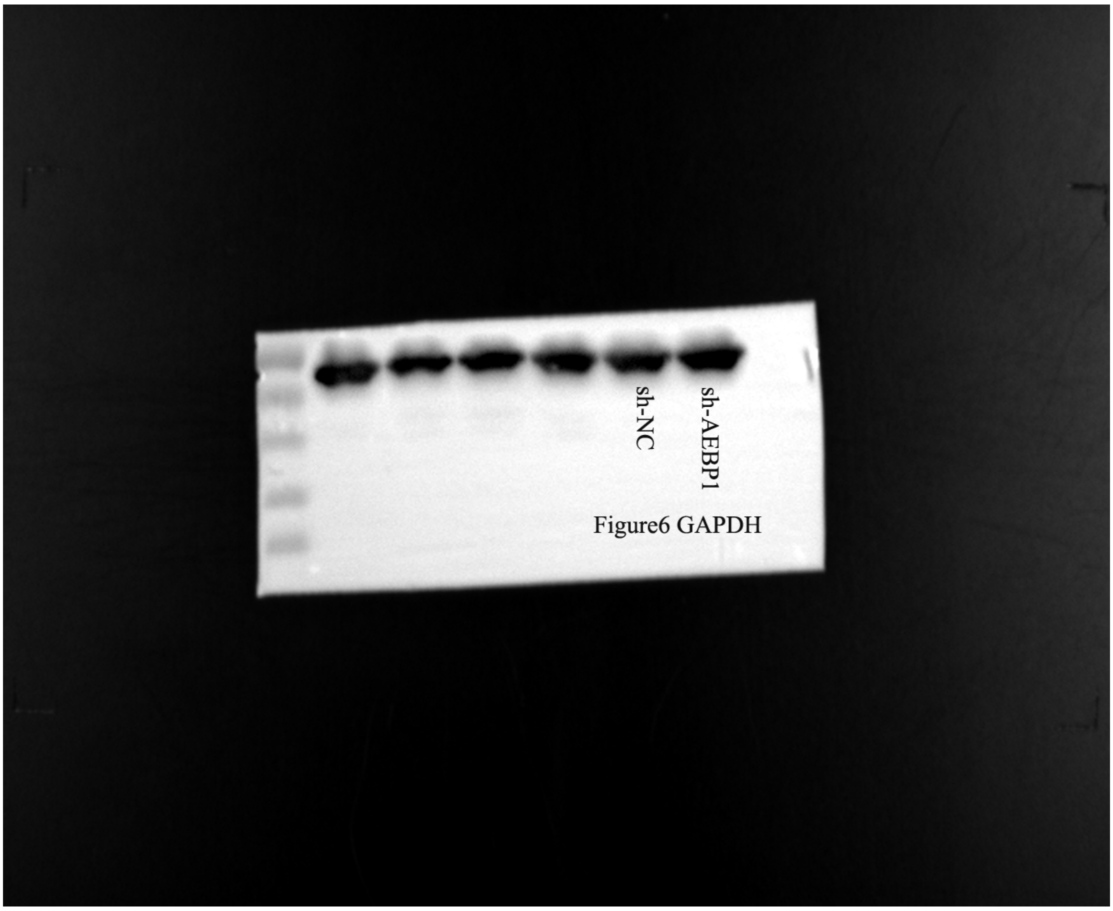
sh-NC
sh-AEBP1
Figure6 GAPDH

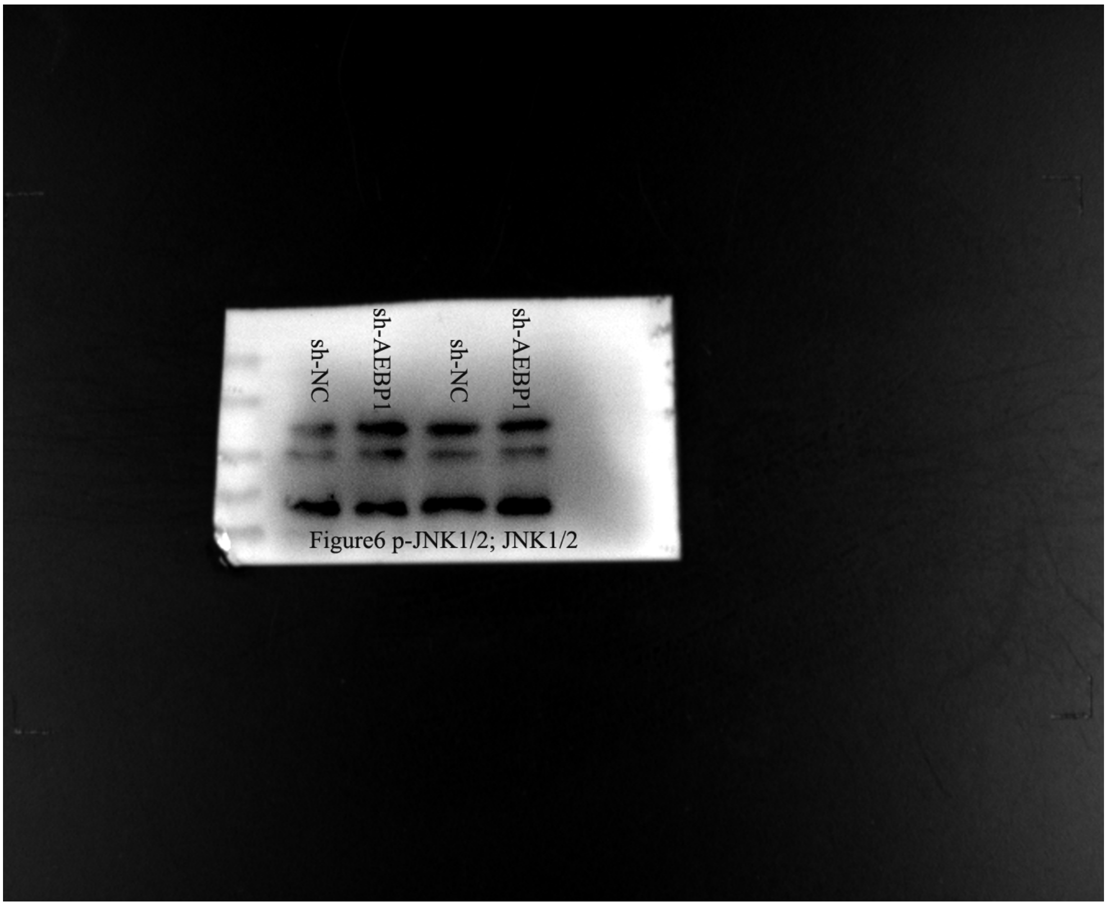

Figure6 p-JNK1/2; JNK1/2

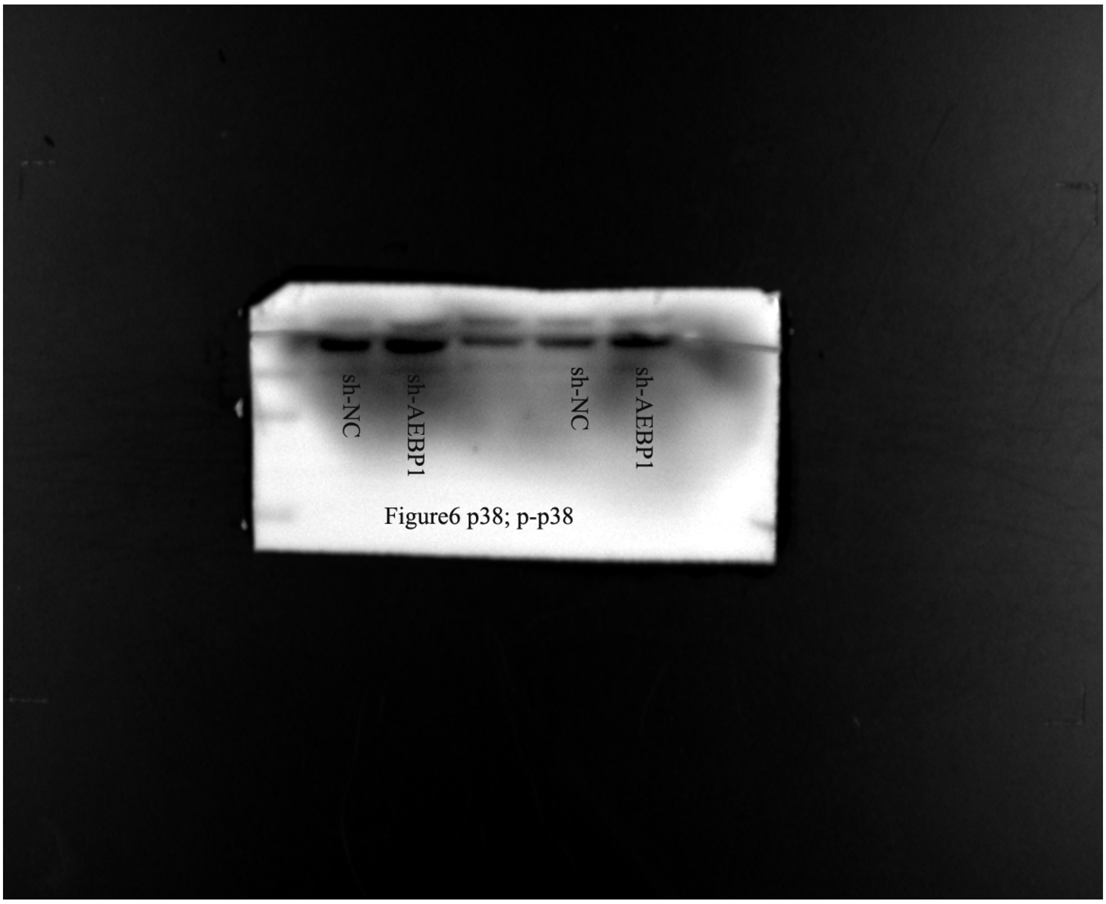
sh-NC
sh-AEBP1
sh-NC
sh-AEBP1
Figure6 p38; p-p38
